# The Catalytic Cycle of the Antioxidant and Cancer-Associated Human NQO1 Enzyme: Hydride Transfer, Conformational Dynamics and Functional Cooperativity

**DOI:** 10.3390/antiox9090772

**Published:** 2020-08-20

**Authors:** Ernesto Anoz-Carbonell, David J. Timson, Angel L. Pey, Milagros Medina

**Affiliations:** 1 Departamento de Bioquímica y Biología Molecular y Celular, Facultad de Ciencias, Instituto de Biocomputación y Física de Sistemas Complejos (GBsC-CSIC and BIFI-IQFR Joint Units), Universidad de Zaragoza, 50009 Zaragoza, Spain; eanoz@unizar.es; 2 School of Pharmacy and Biomolecular Sciences, The University of Brighton, Brighton BN2 4GJ, UK; D.Timson@brighton.ac.uk; 3 Departamento de Química Física, Unidad de Excelencia de Química Aplicada a Biomedicina y Medioambiente, Facultad de Ciencias, Universidad de Granada, 18071 Granada, Spain

**Keywords:** antioxidant enzyme, antioxidant response, cancer, oxidoreductase, enzyme kinetic analysis, functional cooperativity, hydride transfer, kinetic isotope effects, quantum tunneling, conformational dynamics

## Abstract

Human NQO1 [NAD(H):quinone oxidoreductase 1] is a multi-functional and stress-inducible dimeric protein involved in the antioxidant defense, the activation of cancer prodrugs and the stabilization of oncosuppressors. Despite its roles in human diseases, such as cancer and neurological disorders, a detailed characterization of its enzymatic cycle is still lacking. In this work, we provide a comprehensive analysis of the NQO1 catalytic cycle using rapid mixing techniques, including multiwavelength and spectral deconvolution studies, kinetic modeling and temperature-dependent kinetic isotope effects (KIEs). Our results systematically support the existence of two pathways for hydride transfer throughout the NQO1 catalytic cycle, likely reflecting that the two active sites in the dimer catalyze two-electron reduction with different rates, consistent with the cooperative binding of inhibitors such as dicoumarol. This negative cooperativity in NQO1 redox activity represents a sort of half-of-sites activity. Analysis of KIEs and their temperature dependence also show significantly different contributions from quantum tunneling, structural dynamics and reorganizations to catalysis at the two active sites. Our work will improve our understanding of the effects of cancer-associated single amino acid variants and post-translational modifications in this protein of high relevance in cancer progression and treatment.

## 1. Introduction

NAD(P)H quinone oxidoreductase 1 (NQO1; DT-diaphorase; EC 1.6.5.2) is a multi-functional and stress-inducible flavoprotein whose activity is associated with different pathologies, particularly with cancer [1,2]. NQO1 has a wide range of substrates and enzymatic functions associated with antioxidant defense and cancer development, including the NAD(P)H-dependent two-electron reduction of quinones to form hydroquinones, thus avoiding the formation of highly reactive and cytotoxic semiquinones [3,4], the maintenance of vitamin K_3_ and ubiquinone in their reduced state [5,6,7,8] and scavenging of superoxide anions [9]. NQO1 activity is also required for the activation of cancer prodrugs [10,11,12,13,14]. In addition, NQO1 is capable of interacting with over forty different proteins, such as p53, p73α and HIF-1α, and the general effect of these interactions is the protection of protein partners against proteasomal degradation [4,15,16].

NQO1 is upregulated transcriptionally in response to stress [17,18,19,20]. The activation of the antioxidant response induces the expression of NQO1, as well as that of enzymes involved in NADPH generation [21,22,23,24,25]. The expression of NQO1 can also be induced by some antioxidants (such as resveratrol) through the antioxidant response pathway [26,27].

Changes in the activity of NQO1 are associated to different pathologies, including cancer and cardiovascular and neurodegenerative diseases [2]. Intriguingly, the role of NQO1 in cancer development seems to be twofold. NQO1 is overexpressed in certain types of cancer and its inhibition by dicoumarol (Dic), and analogs thereof, arrests the growth of certain cancer cell lines [28,29]. Its overexpression may also contribute to tumor growth through the stabilization of HIF-1α, a master regulator of angiogenesis, thus critical in cancer development [16]. Conversely, cancer cell lines that express inactivating and destabilizing NQO1 polymorphic variants (such as p.P187S and p.R139W) are resistant to certain cancer treatments and are associated with increased cancer risk and poor prognosis [2,30,31,32].

**Figure 1:**
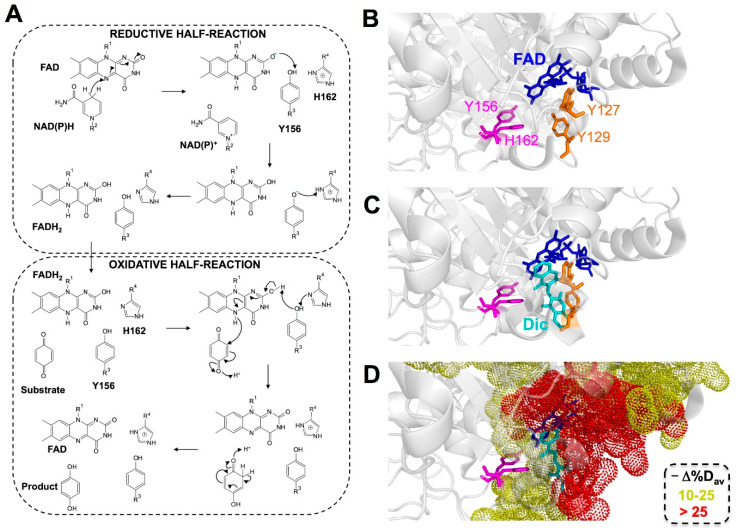
NQO1 catalytic mechanism and protein dynamics. (**A**) Plausible mechanism for the reductive and oxidative half-reactions (for details see the main text). FADH2 undergoes keto/enol tautomerism. Here, we show the initial and final structures of FAD in the enol tautomer. Some reaction steps require the enol or enolate tautomer. (**B**) Changes in NQO1 structural dynamics upon Dic binding from hydrogen-deuterium exchange (HDX) analysis [42]. The upper panel shows the position of the FAD, Y156 and H162 (involved in the stabilization of FADH2) and Y127 and Y129 (critical for Dic and NAD+ binding). (C and D) Dic binding. (**C**) leads to decreased dynamics in residues covering the whole active site, particularly regarding the inhibitor and the FAD binding sites that may contribute to optimizing HT from NAD(P)H and FADH2. (**D**) Δ%Dav is a simple stability metric that refers to the averaged maximal difference in HDX kinetics between two given ligation states according to [42], and a negative value for this parameter reflects an increase in local stability for a given protein segment upon ligand binding (i.e., either HDX is slower and/or its amplitude is reduced upon ligand binding, thus reflecting a locally stabilizing effect upon ligand binding). Note that residue numbering follows the full-length sequence of the protein.

Structurally, NQO1 forms functional homodimers in which each monomer has a two-domain structure, with a large N-terminal domain (approximately residues 1–225) that contains most of the active site and a tightly bound FAD molecule and C-terminal domain (approximately residues 225–274) that complete the active site (i.e., the NAD(P)H and substrate binding sites) and the monomer:monomer interface [33,34,35,36,37]. The enzymatic cycle of NQO1 follows a ping-pong bi-bi mechanism that can be grossly divided into two half-reactions, each of them containing ligand binding/release events, as well as hydride transfer (HT) reactions [2,38,39] (Figure 1A). First, in the reductive half-reaction, NAD(P)H binds to holo-NQO1 (the bound flavin is not released during the enzymatic cycle) and reduces FAD to FADH_2_, thus releasing NAD(P)^+^. This step is fast, with a second-order rate constant in the order of 10^5^–10^6^ M^−1^·s^−1^. It has been proposed that the reduction of FAD by NAD(P)H) occurs by direct HT between them, likely leading to the formation of FADH_2_ in the enolate form. This tautomer would have a negatively charged O2F that may become stabilized upon sequential proton transfer with the side chains of Y156 and H162. In the oxidative half-reaction, the substrate binds and is reduced by FADH_2_ (this half-reaction is much faster, with a second-order rate constant of 10^9^ M^−1^·s^−1^), thus releasing the reduced substrate and regenerating the holo-enzyme [2,34]. In this second half-reaction, the charge/proton transfer between FADH_2_, Y156 and H162 would likely occur in the reverse sense [40,41].

Importantly, most of our knowledge on the NQO1 kinetic mechanism has come from analysis of either ligand binding/release events from crystallographic analyses, or from single-wavelength kinetic analyses focused on the changes in FAD spectral properties associated with chemical steps (i.e., HT) [34,38,40,43,44]. Several studies have supported that changes in molecular dynamics throughout the catalytic cycle should also be considered [42,45]. The use in these studies of Dic, a potent competitive inhibitor of NAD(P)H [6,39], has supported the proposal that NAD(P)H binding may cause minimal structural changes [46,47] but affects the stability and structural dynamics of the active site, which contributes to the enhancement of catalysis by reducing the reaction free energy barrier(s) and/or promoting quantum tunneling effects [42,45] (Figure 1B). Interestingly, Dic binding might also allow for the communication of local stability effects between active sites during the catalytic cycle [42,48,49]. The existence of functional and structural non-equivalence between the active sites may also explain the apparent negative cooperativity found for Dic binding (mainly reflected by inhibition studies with the holo-protein) [48,49]. A critical role of protein dynamics in the activity and stability of NQO1 is further supported by the study of the inactivating and destabilizing effects of the cancer-associated p.P187S polymorphism [30,31,50,51,52,53]. Importantly, long-range communication of stability effects due to inactivating mutations and polymorphisms, as well as due to the presence of suppressor mutations and ligand binding (Figure 1B), seems to be a general phenomenon in NQO1 [30,33,41,42,50,54,55,56,57].

A deep understanding of the kinetic mechanism of this important metabolic and antioxidant enzyme would allow us to rationalize the effects of missense variants and enable the improved design of inhibitors and drugs which are activated by NQO1 as chemotherapeutics [58,59,60,61,62,63]. However, to the best our knowledge, no study has investigated the NQO1 kinetic mechanism from an integrated perspective of the changes occurring in the NQO1 structure and dynamics that facilitate HT reactions. We provide here such an integrated perspective on the NQO1 catalytic mechanism by kinetically evaluating the different events occurring in the reductive and oxidative half-reactions by using stopped-flow spectrophotometry with photodiode detection combined with temperature-dependent kinetic isotopic effects (KIEs) [64]. Overall, our results show that HT from NAD(P)H to FAD occurs through two different pathways with widely different kinetics, likely reflecting that the two active sites in the NQO1 dimer are not equivalent. This non-equivalence may explain the binding cooperativity observed for Dic. In addition, these two pathways largely differ in the contributions from structural and vibrational dynamics along the reaction coordinate(s). Thus, this work constitutes an important advance in deciphering the dynamics at the active site of this structurally complex enzyme during catalysis. Our work will help in the rational design of more potent and specific mechanism-based NQO1 inhibitors, as well as to understand the functional consequences of naturally occurring NQO1 missense variants and post-translational modifications and the structural and energetic basis of functional cooperativity in this enzyme.

## 2. Materials and Methods

### 2.1. Materials

All the chemicals were purchased with high purity (typically > 99%) from Sigma-Aldrich and Merck, and these were used without further purification, unless otherwise indicated. The 5-deazariboflavin was a gift from the G. Tollin Lab (University of Arizona). The stereospecifically labeled nicotinamide nucleotide [4R-^2^H_1_]-NADH (with the deuterium in the A face of the nicotinamide ring, NADD) was synthetized enzymatically using [^2^H_8_]-propanol/alcohol dehydrogenase following previously described protocols [65]. Milli-Q water was obtained from a Milli-Q^®^ Reference water purification system (Millipore, Madrid, Spain) and used for the preparation of all buffers and media.

### 2.2. Protein Expression and Purification

The wild-type (WT) NQO1 cDNA was cloned into a pET-46 Ek/LIC vector [31]. This plasmid was used to transform BL21(DE3) cells (Agilent) for protein expression. Transformed cells were grown in 240 mL of autoclaved Luria–Bertani (LB) medium containing 0.1 mg·mL^−1^ ampicillin (Canvax Biotech) (LBA) overnight at 37 °C. These cultures were diluted into 4.8 L of LBA and grown at 37 °C for 3 h under shaking (at 150–160 rpm) to reach an optical density of about 0.6. Then, cultures were cooled down to 25 °C and induced with 0.5 mM isopropyl β-D-1-thiogalactopyranoside (IPTG, Canvax Biotech). After 4 h, cells were harvested by centrifugation (8000 *g*, 15 min, 4 °C) and cells were frozen and maintained at −80 °C overnight. Cells were resuspended in binding buffer (BB; 20 mM NaH_2_PO_4,_ 300 mM NaCl, 50 mM imidazole; pH was adjusted to 7.4 using concentrated HCl) plus 1 mM phenylmethylsulfonyl fluoride (PMSF), sonicated in an ice bath and centrifuged at 24,000 *g* for 20 min at 4 °C. Supernatants (soluble extracts) were loaded into immobilized-metal affinity chromatography (IMAC) columns (His Gravitrap, Ni Sepharose 6 fast flow resin, 1 mL bed volume, GE Healthcare), washed with 25 volumes of BB and eluted with 2.5 mL of BB containing 500 mM imidazole (pH was adjusted to 7.4 by addition of concentrated HCl). These eluates were buffer exchanged using PD-10 columns (GE Healthcare) to the storage buffer (50 mM HEPES-KOH pH 7.4), frozen in liquid nitrogen and stored at −80 °C. Typically, this procedure yielded about 20 mg of purified NQO1 protein.

**Figure 2:**
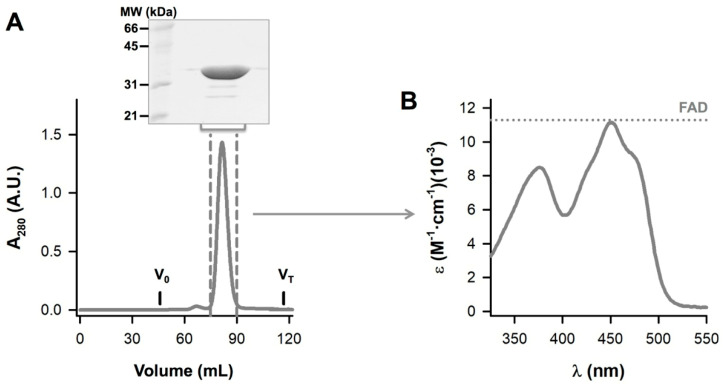
Purification of NQO1. (**A**) Size-exclusion chromatography (SEC) chromatogram of NQO1 protein. About 20 mg of protein from IMAC (immobilized-metal affinity chromatography) were injected into a HiLoad^®^ 16/600 Superdex^®^ 200 pg (GE Healthcare) running on 20 mM HEPES-NaOH, 200 mM NaCl pH 7.4 at 20 °C. Void (V_0_) and total (V_T_) volumes are indicated. Fractions eluted between 75 and 90 mL were pooled and concentrated. The purity was checked by SDS-PAGE in 12% acrylamide gels (inset). (**B**) Concentrated protein was exchanged to HEPES-KOH 50 mM pH 7.4 and the UV–visible absorption spectrum was collected at a 20 μM protein concentration. The extinction coefficient of free FAD is indicated for sake of comparison.

About 20 mg of protein from IMAC were thawed, centrifuged for 10 min at 24,000 *g* and 4 °C, diluted in storage buffer to a final volume of 5.5 mL and 5 mL of this protein solution were injected into a HiLoad^®^ 16/600 Superdex^®^ 200 pg (GE Healthcare). Size-exclusion chromatography (SEC) was carried out using 20 mM HEPES-NaOH, 200 mM NaCl pH 7.4 as a mobile phase at 20 °C and using a 1.5 mL·min^−1^ flow rate. Void (V_0_) and total (V_T_) volumes were determined using blue dextran and acetone, respectively (Figure 2A). Fractions eluting between 75 and 90 mL were pooled, concentrated using Amicon^®^ Ultra-15 Centrifugal Filter Units-30,000 NMWL (Millipore), mixed with a final concentration of 1 mM FAD and exchanged to HEPES-KOH 50 mM pH 7.4 using PD-10 columns. The UV–visible absorption spectra of the purified protein were collected at a 20 μM protein concentration using 1-cm pathlength quartz cuvettes on a Cary 50 or 100 spectrophotometer (Agilent). Protein concentration and FAD content were determined from the absorbance at 280 nm and 450 nm, respectively [50]. Briefly, the experimental spectrum was converted into molar extinction units (M^−1^·cm^−1^) using the absorbance at 280 nm considering that the extinction coefficient of NQO1 monomer with bound FAD is the sum of its intrinsic protein extinction coefficient (47,900 M^−1^·cm^−1^, based on its amino acid sequence) *plus* the contribution from bound FAD (22,000 M^−1^·cm^−1^ ·fraction of bound FAD per monomer) [31]. The former contribution can be assessed experimentally for each spectrum from the absorbance at 450 nm (ε_450_ = 11,300 M^−1^·cm^−1^) [66]. As we have previously indicated [50], this approach considers that the spectral properties of FAD are similar when bound to NQO1 to those of the free ligand. NQO1 samples obtained from different purifications contained 0.97–0.99 moles of FAD per mole of NQO1 monomer as assessed by this procedure (Figure 2B).

### 2.3. NQO1 Redox Properties Evaluated by Absorption Spectroscopy

Photoreduction of NQO1 was achieved by irradiating the oxidized protein (NQO1_ox_) under anaerobic conditions in the presence of 2 mM EDTA and 8 µM 5-deazariboflavin [67]. Experiments were performed in HEPES-KOH, pH 7.4 at 25 °C in home-made spectrophotometer cuvettes. Glucose (at a 310 mM final concentration) and glucose oxidase (at a 10 units·mL^−1^ final concentration) were added to all the solutions to remove trace amounts of oxygen. The stepwise reduction of the protein was achieved by light irradiation from a 250 W slide projector for different periods of time, for which the UV–visible spectrum was then recorded in a Cary 100 spectrophotometer (Agilent). Once fully reduced (no variation in the UV–visible spectra despite further irradiation), the protein was re-oxidized by breaking the anaerobic conditions and exposure to atmospheric air, and absorption spectra were recorded until complete re-oxidation.

The ability of NAD^+^ to re-oxidize the hydroquinone form of the protein (NQO1_hq_) was evaluated by using anaerobic solutions of photoreduced NQO1 (7.5 µM), produced by following the above described procedure in specially designed cuvettes. NQO1_hq_ was them mixed with an NAD^+^ solution placed in the same cuvette lateral arm, providing a final 1:1 protein:coenzyme ratio and the kinetics of the re-oxidation of the enzyme were followed in the full protein spectral range using an Agilent 8453 photodiodearray spectrophotometer (Agilent).

### 2.4. Stopped-Flow Pre-Steady-State Kinetic Measurements

Fast HT reactions from NAD(P)H/D to NQO1_ox_, as well as from NQO1_hq_ (generated by NQO1_ox_ mixed with NADH at stoichiometric ratio) to 2,6-dichlorophenol indophenol (DCPIP), were measured using a stopped-flow spectrophotometer from Applied Photophysics (SX.18MV, Applied Photophysics Ltd., Leatherhead, UK) interfaced with a photodiode array detector and under anaerobic conditions, following previously established protocols [68,69]. All samples were made anaerobic (in specially designed tonometers by successive evacuation and O_2_-free argon flushing) before introduction into the stopped-flow syringes. NQO1_ox_ (7.5 μM) was mixed with NADH/D at concentrations ranging from 1:1 to 1:14 NQO1_ox_:NADH/D ratios, while when using NADPH, the single 1:1 ratio was used. To evaluate re-oxidation, NQO1_hq_ (7.5 μM) was mixed with stoichiometric amounts of DCPIP. Additionally, Dic was used as an inhibitor of both the reductive and oxidative half-reactions, adding it at 1:1 and 1:4 NQO1: Dic ratios. Reactions were studied in 20 mM HEPES-KOH, pH 7.4 with glucose/glucose oxidase (310 mM/10 units·mL^−1^), at 25 °C and/or 6 °C. Multiple wavelength absorption data in the flavin absorption region (400-900 nm) were collected and processed using the ProData-SX software (Applied Photophysics Ltd.). Time-dependent spectral deconvolution was performed by global analysis and numerical integration methods using Pro-Kineticist (Applied Photophysics Ltd.). Collected data were fitted to either single- or multi-step (A→B→n….→Z) models allowing for estimation of the corresponding observed conversion rate constants (*k*_obsA→B,_ *k*_obsB→C_, …) at each NAD(P)H/D concentration, as well of the spectra of intermediate and final species [70]. A, B, n and Z are *spectral species*, reflecting a distribution of enzyme species at any time throughout the course of the enzyme: coenzyme interaction, including HT (or deuteride transfer, DT) or reorganization processes, and do not necessarily represent a single distinct enzyme intermediate. Since none of them represents individual species, their spectra cannot be included as fixed in the global fitting.

The *k*_obs_ values showing hyperbolic dependence profiles on the NAD(P)H/D concentration were fitted to a function (1) that describes binding at a single site followed by reorganization or HT/DT processes, allowing for the determination of the corresponding equilibrium constant (*K*_d_), as well as the rate constant for the subsequent process (*k*) [69,70]:

(1) $$k_{obs}=k_{A\to B}=k_{B\to C}=\frac{k\cdot \left[NAD\left(P\right)H\right]}{\left[NAD\left(P\right)H\right]+K_{d}}$$

Depending on the process, *k* might account for the rate constant of the rate-limiting step for complex formation, *k*_on_, or for the HT/DT rate constant, *k*_HT_ or *k*_DT_. *K*_d_ might respectively account for the complex dissociation constant, $K_{d}^{NADH/D}$, or for a *reorganization* constant related to the transition between reaction intermediate species, $K_{d}^{reg}$.

### 2.5. Kinetic Isotopic Effects (KIEs)

For the estimation of primary kinetic isotopic effects in the HT process [68], HT or DT observed rate constants (^HT^*k*_obs_ or ^DT^*k*_obs_) from NADH/D to NQO1_ox_ were evaluated at different temperatures in the 5.3−20 °C range, in samples containing equimolecular mixtures (7.5 μM of each component) using NADH and [4R-^2^H]-NADD, unless otherwise indicated.

Kinetic isotope effects (KIEs) on rate constants were calculated as follows:

(2) $$\mathrm{KIE}=\frac{k_{HT}}{k_{DT}}=\frac{{}^{HT}k_{obs}}{{}^{DT}k_{obs}}$$

For each isotope, the fitting of the observed rates to the Arrhenius equation was calculated:

(3) $$k=A*e^{-\frac{Ea}{RT}}$$

providing the values corresponding to Arrhenius pre-exponential (frequency) factors (*A*_H_ and *A*_D_) and activation energies (*E*_aH_ and *E*_aD_). The temperature dependence of the KIE was analyzed by combining Equations (2) and (3). Additionally, the activation enthalpies (ΔH^‡^) and entropies (ΔS^‡^) were calculated using the Eyring equation:

(4) $$\ln\left(\frac{k_{obs}}{T}\right)=\ln\left(\frac{k_{B}}{h}\right)+(\Delta S^{‡}/R)-\left(\frac{\Delta H^{‡}}{R\cdot T}\right)$$

where *k*_B_ is the Boltzmann constant (1.3806·10^−23^ J·K^−1^) and *h* is the Planck constant (6.626·10^−34^ J·s).

## 3. Results and Discussion

### 3.1. Human NQO1 Does Not Stabilize Intermediate Semiquinone States upon Photoreduction

**Figure 3:**
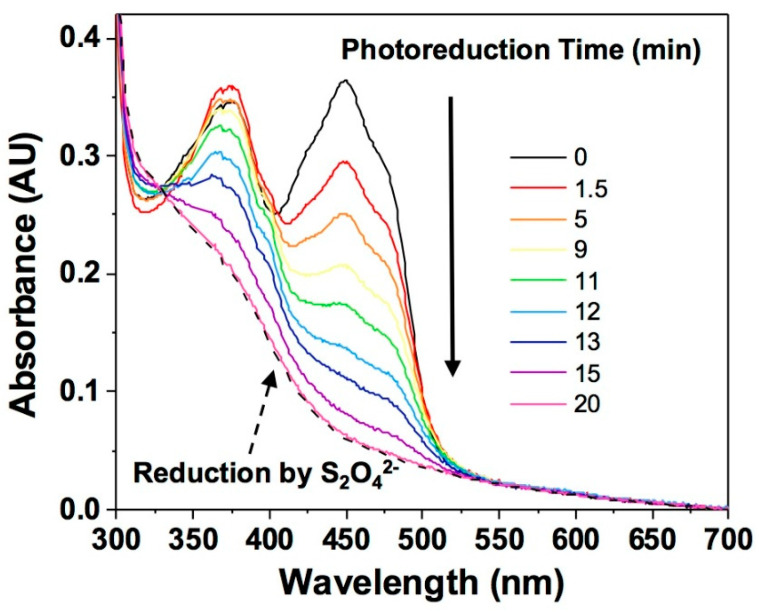
Photoreduction of NQO1. Colored spectra correspond to different illumination time points throughout the photoreduction process. The dashed black line indicates the spectra corresponding to a sample chemically reduced by dithionite (S_2_O_4_^2−^).

NQO1_ox_ exhibits the characteristic UV–visible spectra of flavoproteins, with maxima at 278, 375 and 449 nm, and shoulders at 422 and 475 nm (Figure 3). Upon photoreduction, the FAD cofactor exists in the hydroquinone state, NQO1_hq_ (i.e., a two-electron reduction), as denoted by the decrease in absorbance at 370 and 450 nm. Full reduction was achieved after 15 min of irradiation, with equivalent spectral features to those observed upon reduction with an excess of sodium dithionite (Figure 3). The photoreduction occurs without the appearance of any red-shifted absorbance band, indicative of the stabilization of the FAD blue-neutral semiquinone, but subtle changes in absorbance at 375, 400 and 480 nm might point to traces of the red-anionic semiquinone radical [71], as observed for other oxidases, including rat liver NQO1 [44]. Such a lack of semiquinone intermediates indicates that reduction of the semiquinone to the hydroquinone state is thermodynamically more favorable and kinetically faster than the reduction of the oxidized to the semiquinone species [72]. This observation agrees with the absence of detectable semiquinone paramagnetic signals when evaluating the redox cycle of the enzyme and with mammalian quinone oxidoreductases, which are a notable exception in that they only function by a compulsory two-electron transfer [73]. Such observations denote a less negative midpoint reduction potential of the FADH·/FADH_2_ couple with respect to the FAD/FADH· one. Finally, upon mixing the photoreduced protein with atmospheric air, the initial absorbance spectrum of the oxidized protein was restored (not shown).

### 3.2. The Catalytic Cycle of NQO1

To evaluate the kinetics of the reductive and oxidative half-reactions in the catalytic cycle of NQO1 (Figure 1A), we studied the spectral changes occurring in the band-I (400–500 nm range) of the flavin by using fast kinetics stopped-flow spectrophotometry.

#### 3.2.1. Non-Equivalent Active Sites in the NQO1 Dimer throughout the Reductive Half-Reaction

**Figure 4:**
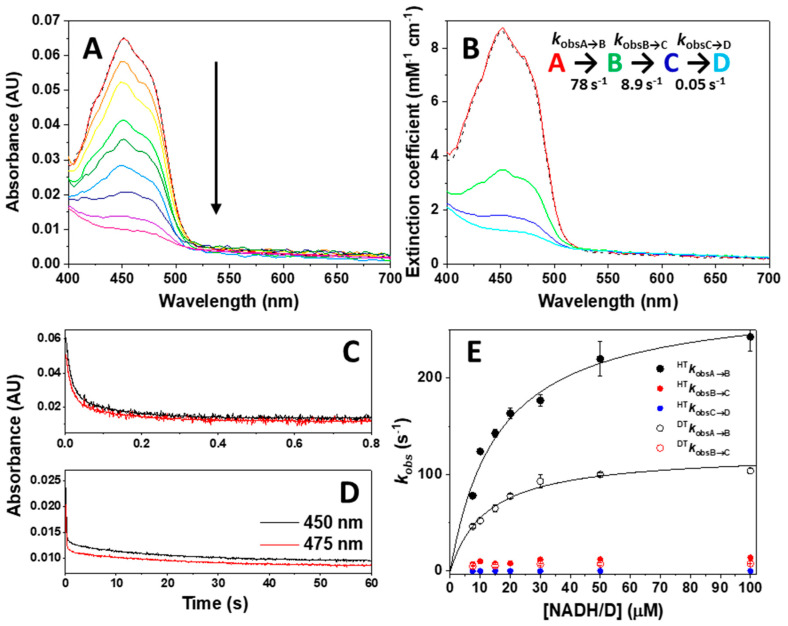
Kinetics of the NQO1 flavin reduction by NADH/D. (**A**) Spectral evolution on a 0−60 s timescale after mixing NQO1_ox_ (7.5 μM) with NADH (7.5 µM) in 20 mM HEPES-KOH, pH 7.4, at 6 °C. Different colored lines correspond to the spectra at different reaction times. (**B**) Spectral deconvolution of intermediate species observed during the reaction when fitting to a four-state model and the corresponding calculated observed rate constants. In panels A and B, the dashed line represents the spectrum of NQO1_ox_ before mixing. (**C**,**D**) Decay of kinetic traces at 450 nm and 475 nm and fittings to the model. (**E**) Dependence of *k*_obs_ values on the NADH/D concentration. The trace for the fitting to Equation (1) for *k*_obsA__→B_ values is shown as a black line. Error bars correspond to the SD for at least three different replicates. Spectral evolution (**A**) and deconvolution (**B**) are from a single measurement and representative from n > 3.

We first evaluated the kinetics of the reductive half-reaction by mixing NQO1_ox_ with NADH at 6 °C under strict anaerobic conditions. A decrease in the absorption at the FAD band-I was quickly observed without detection of semiquinone traces (Figure 4A), in agreement with FAD reduction to the hydroquinone form. The final spectrum after the overall HT compares well with the fully photoreduced NQO1 and dithionite reduced spectra, suggesting full reduction of the cofactor was achieved (Figure 3 vs. Figure 4A). This observation envisages a less negative reduction potential of the NQO1 FAD/FADH_2_ pair regarding the NAD^+^/NADH redox pair (*E*’_0_ = −320 mV) [74] under our experimental conditions. This agrees with the value of −159 mV previously reported for the rat enzyme [44].

To provide further insight into the mechanism of the reductive half-reaction, we submitted these kinetic data to global spectral deconvolution (Figure 4B). Several mechanisms were evaluated, but only a minimal three-step non-reversible mechanism (A→B→C→D) (Figure 4B–D) reproduced the experimental data well. The lack of major spectral changes in the instrumental dead time suggested that species A corresponds with the initial mixing of NQO1_ox_ and NADH. The conversion of species A into B was very fast (initial ~10–40 ms of reaction) and contributed to nearly 75% of the decay of the band-I absorption (Figure 4B). Therefore, this step comprises the HT process from NADH to FAD and its reduction. The observed rate constants for this process, *k*_obsA→B_, showed hyperbolic dependence on NADH concentration (Figure 4E), allowing for the determination of a NADH dissociation constant (*K*_d_^NADH^), as well as of a limiting HT rate constant (*k*_HT1_), without considering the occurrence of any reverse HT reaction. The limiting value for *k*_HT1_ of 284 ± 17 s^−1^ (at 6 °C) indicates a very fast HT from NADH to NQO1_ox_, which compares well with the steady-state catalytic constant (*k*_cat_) of 180–200 s^−1^ (at 30–37 °C) [31,33,48]. This agreement supports that the HT process is the rate-limiting step within the reductive half-reaction. Alternative scenarios, in which substrate binding and/or product release could be rate-limiting steps are discussed in Appendix A. Kinetic analysis on the A→B process also allows us to determine a *K*_d_^NADH^ of 16 ± 3 µM, a value 10-times lower than the reported *K*_M_^NADH^ (160–240 µM, using DCPIP as substrate) [31,33,48]. This observation indicates a tighter interaction in the reactive NQO1_ox_:NADH complex than in the subsequent complexes formed throughout the reaction, and justifies the lack of spectroscopic detection of charge–transfer complexes (CTCs).

This initially observed HT step (A to B) was followed by another process (B to C) that led to the nearly full reduction of the flavin bound to NQO1. In fact, this process essentially accounted for the remaining 25% of the total absorption decrease at the flavin band-I, and, therefore, must also be related to an HT process. The *k*_obsB→C_ values were considerably slower than those for *k*_obsA→B_, and roughly dependent on the coenzyme concentration (Figure 4E), providing a limiting rate for this second HT event, *k*_HT2_, in the 10–15 s^−1^ range. Our kinetic analysis showed a final step to achieve full FAD reduction (C to D), but this process accounted for a very small and slow spectroscopic change (with rate constants 5000 times slower than for the A→B process), suggesting that it might not be of catalytic relevance.

**Figure 5:**
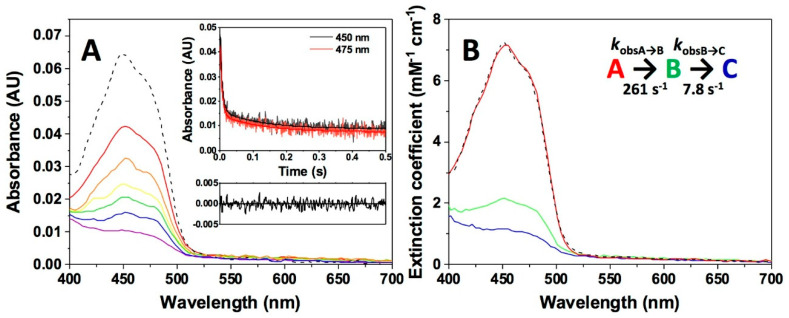
Kinetics of the NQO1 flavin reduction by NADPH. (**A**) Spectral evolution on a 0–0.5 s timescale after the mixing NQO1_ox_ (7.5 μM) with NADH (7.5 µM) in 20 mM HEPES-KOH, pH 7.4, at 6 °C. Different colored lines correspond to the spectra at different reaction times. The inset shows the decay of kinetic traces at 450 nm and 475 nm, the fittings to the model, and the residuals at 450 nm to show the quality of the fitting. (**B**) Spectral deconvolution into the different species observed along the reaction from fittings to a two-step model and calculated rate constants. Spectral evolution (**A**) and deconvolution (**B**) are from a single measurement and representative from n > 3.

NQO1 can also use NADPH as electron donor, which is a better reductant than NADH (with six-fold higher catalytic efficiency and a four-fold enhancement in *k*_cat_, [31,34,75]). Consequently, NQO1_ox_ mixed with NADPH at a 1:1 ratio shows a faster FAD reduction than when using NADH, part of which occurs in the instrumental dead time (Figure 5A). The process was best described as a two-step process (A→B→C) (Figure 5B), with at least 80% of flavin reduction occurring in the first step. The *k*_obsA→B_ and *k*_obsB→C_ values at stoichiometric concentrations were 261 ± 13 and 7.8 ± 0.3 s^−1^, respectively, with *k*_obsA→B_ being 3.7 times faster than when using the same NADH ratio, while *k*_obsB→C_ values resulted in the same range. Hence, the overall HT process is faster when NADPH is the hydride donor, consequently preventing studies using higher coenzyme concentrations.

**Figure 6:**
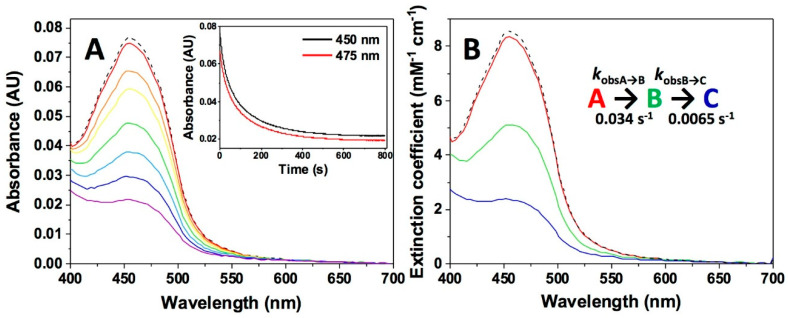
Kinetics of NQO1 flavin reduction by NADH in the presence of the Dic inhibitor. (**A**) Spectral evolution after the mixing in the stopped-flow equipment of NQO1_ox_ (7.5 μM) with NADH (7.5 µM) in the presence of Dic (7.5 µM) in 20 mM HEPES-KOH, pH 7.4, at 6 °C on a 0–800 s timescale. Different colored lines correspond to the spectra at different reaction times. The inset shows the decay of kinetic traces at 450 nm and 475 nm, as well as the fitting to a three-state model. (**B**) Spectral deconvolution of intermediate species observed during the reaction upon fitting to a two-step model and calculated observed rate constants. In panels A and B, the dashed line represents the protein spectrum before mixing. Spectral evolution (**A**) and deconvolution (**B**) are from a single measurement and representative from n > 3.

Altogether, and considering that NQO1_ox_ is a dimer, these data allowed us to suggest that the reduction of each protomer within the dimer might occur at very different rates. We must note that, according to the change in the flavin band-I magnitudes for the A→B and B→C steps, a part of the reduction of the *slower protomer* might occur within the first A→B step. To test this hypothesis, we used Dic to slow down the reductive half-reaction of NQO1 by NADH [6,39]. Dic competes for the NAD(P)H binding site, blocking access to the nicotinamide part of the coenzyme and, as a consequence, preventing the HT from the nicotinamide to the flavin cofactor [46]. As shown in Figure 6, the presence of Dic causes a considerable slowdown of the overall HT processes by NADH. Moreover, the spectral evolution was best described by a two-step mechanism (A→B→C), with each of the two steps accounting for half of the spectral change corresponding to FAD reduction. The full observation of both individual processes was likely possible because of the considerable reduction in *k*_obsA→B_ and *k*_obsB→C_ caused by Dic, respectively 0.034 ± 0.003 and 0.0065 ± 0.0005 s^−1^ at stoichiometric concentrations, which implies a ~2000-fold decrease. Higher Dic ratios (1:1:4 protein/NADH/dicoumarol) produced further slowdowns of both steps (Table 1), while increasing NADH concentrations (50 µM, 1:6.6:4 of protein/NADH/Dic) only slightly increased reduction rate constants, hardly preventing Dic inhibition. Such observations are easily explained by the higher affinity of three orders of magnitude of Dic vs. NADH (*K*_d_^Dic^ typically in the 1−20 nM range; [33,49,55] and Section 3.2.1). Therefore, these inhibition studies with Dic strongly supported that the reduction of the FAD cofactor at the two active sites of the NQO1 dimer occurs at different rates.

**Figure 7:**
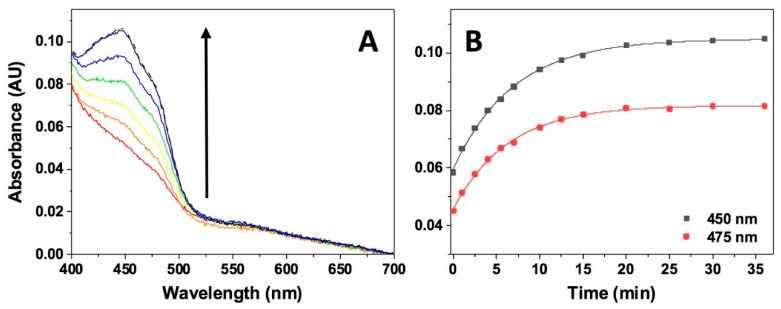
Kinetics of the reaction of NQO1_hq_ with NAD^+^. (**A**) Spectral evolution after mixing (in an anaerobic cuvette) NQO1_hq_ (7.5 μM)) with a 1:1 ratio of NAD^+^ in 20 mM HEPES-KOH, pH 7.0, at 6 °C on a 0–35 min timescale. Different colored lines correspond to the spectra at different reaction times. (**B**) Detail of kinetic traces at 450 nm and 475 nm and fitting to a single exponential function. Spectral evolution (**A**) is from a single measurement and representative from n > 3.

So far, our analyses for the reductive half-reaction of NQO1_ox_ by NAD(P)H have pointed to the reverse HT reactions being practically negligible. To confirm this, we mixed photoreduced NQO1_hq_ with stoichiometric concentrations of NAD^+^, in specially designed spectrophotometer cuvettes and under anaerobic conditions, and followed spectral changes over the time. Once the reaction components were mixed, the FAD cofactor became very slowly re-oxidized without the stabilization of any semiquinone or CTC intermediates (Figure 7). The overall protein re-oxidation resulted in a monophasic process with a rate constant of 0.0025 ± 0.0005 s^−1^. This parameter would relate to the apparent HT rate constant for the backward reaction (^app^*k*_HT-1_), being around 28,000 times slower than the corresponding forward process. Therefore, the equilibrium of the reductive half-reaction is fully displaced towards the production of NQO1_hq_, in agreement with the main physiological role of the enzyme in the detoxification of quinones by their reduction.

#### 3.2.2. Non-Equivalent Active Sites in the NQO1 Dimer throughout the Oxidative Half-Reaction

**Figure 8:**
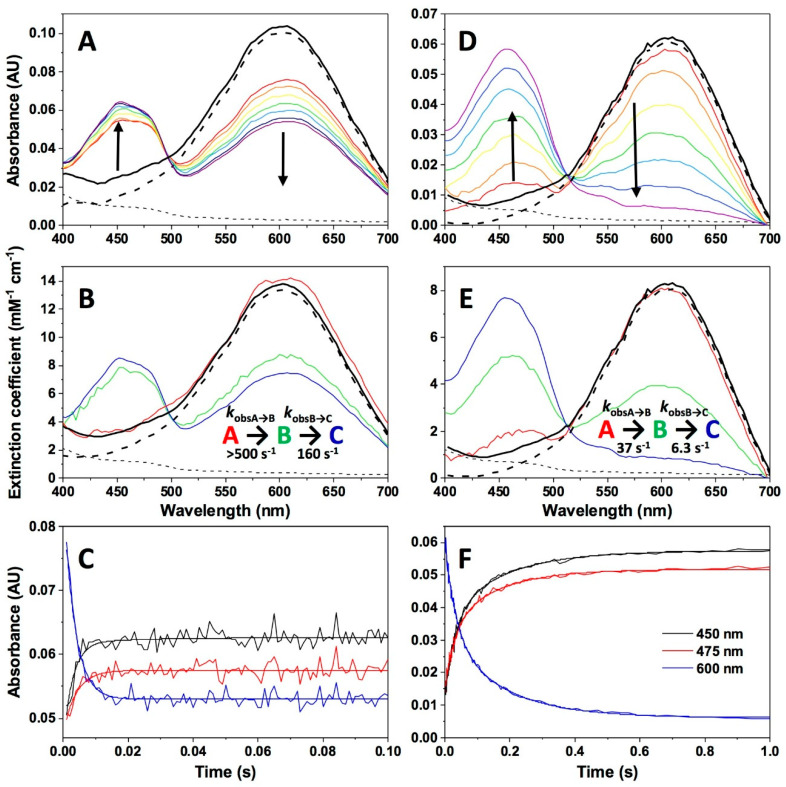
Kinetics of NQO1_hq_ re-oxidation by DCPIP (2,6-dichlorophenol indophenol). (**A**) Spectral evolution after mixing NQO1_hq_ (7.5 μM) with DCPIP (7.5 µM) in 20 mM HEPES-KOH, pH 7.4, at 6 °C on a 0–0.1 s timescale. (**B**) Spectral deconvolution of intermediate species observed during the reaction when using a three-state model. (**C**) Kinetic traces at 450 nm, 475 nm and 600 nm. Experimental data as well as the fitting to the three-state mechanism are shown. (**D**) Spectral evolution after mixing NQO1_hq_ (7.5 μM) with DCPIP (7.5 µM) in the presence of Dic (7.5 µM) in 20 mM HEPES-KOH, pH 7.4, at 6 °C on a 0–1 s timescale. (**E**) Spectral deconvolution of intermediate species obtained from analysis using a three-state model. (**F**) Kinetic traces at 450 nm, 475 nm and 600 nm. Experimental data as well as the fitting to the three-state mechanisms are shown. Dashed lines (panels **A**,**B** and **D**,**E**) correspond to the initial spectra of NQO1_hq_ and DCPIP (bold), the bold black line is the addition of these two spectra (species at t = 0) and the different colored lines correspond to the spectra at different reaction times. Spectral evolution (**A**,**D**) and deconvolution (**B**,**E**) are from a single measurement and representative from n > 3.

Although NQO1 can reduce a wide variety of substrates [4], most of them are not appropriate for enzymatic studies due to their low solubility in aqueous solutions, their characteristic spectral properties or, in some cases, their fast reduction that precludes pre-steady-state kinetic characterization using stopped-flow spectroscopy [34,44]. We have here used DCPIP, a suitable and artificial electron acceptor often used in activity measurements of human NQO1 [4], to study its oxidative half-reaction. Nonetheless, mixing NQO1_hq_ with DCPIP at equimolecular concentrations also resulted in the extremely fast re-oxidation of the protein, even at low temperatures, and with nearly 50% of the spectral changes occurring in the instrumental dead time (Figure 8A–C). The observed overall process was best fitted to a two-step mechanism (A→B→C), with the initial step accounting for most of the spectroscopic changes and exhibiting observed rate constants, *k*_obsA→B_, above the instrumental measurement limit (>500 s^−1^, at stoichiometric reactant ratios). Conversely, the second step (B→C) shows minor, and probably *biased*, contributions both in terms of *k*_obsB→C_ (160 s^−1^) and amplitude. These values for the rate constants in the oxidative half-reaction further reinforce that the reductive half-reaction is rate-limiting in NQO1 catalysis, also preventing additional analyses at higher NQO1_hq_:DCPIP ratios.

Although Dic is usually reported as an inhibitor of the reductive half-reaction, we also evaluated its effect in the oxidative half-reaction. The re-oxidation of NQO1_hq_ by DCPIP is also significantly slowed down in the presence of Dic (1:1 of protein/NADH mixed with 1:1 DCPIP/Dic, Figure 8D,E), which might not be surprising because Dic shares the binding site with both the electron donor and acceptor [46]. The presence of Dic has a considerable effect on both *k*_obsA→B_ and *k*_obsB→C_, which decreased to 38 ± 3 and 6.3 ± 1.2 s^−1^, respectively, at a 1:1 ratio and even more at higher Dic concentrations (Table 1), while the amplitudes of the changes became similar for both processes (Figure 8D–F). These data indicate that the two active sites of NQO1 are also non-equivalent regarding the kinetics of the oxidative half-reaction.

Although the main enzymatic role of NQO1 is associated with the mandatory two-electron reduction of substrates, reactivity with artificial one-electron oxidants, such as ferricyanide, has been widely reported for the characterization of *Saccharomyces cerevisiae* Lot6p and rat and human NQO1 [76,77]. The rapid mixing of reduced NQO1_hq_ with ferricyanide (1:1 reduction equivalent ratio) coursed with the re-oxidation of the FAD cofactor (in ~ 0.4 s) without any trace of semiquinone stabilization and with *k*_obsA→B_ of 219 ± 12 s^−1^ and *k*_obsB→C_ of 29 ± 4 s^−1^, values considerably lower when compared to two-electron substrates.

### 3.3. Dynamics at the NQO1 Active Sites Differentially Contribute to the Two HT Events Representing the Reductive Half-Reaction

We then used fast kinetics measurements to investigate primary KIEs (using [4R-^2^H]-NADD), as well as the temperature dependence of the rate constants and KIEs, in the context of the Arrhenius equation. The resulting parameters may provide information on the structural organization and dynamics at the active site of enzymes during HT catalysis [68]. Due to ^HT^k_obsA→B_ values being close to the instrumental limit upon increasing the coenzyme concentration (see black closed circles in Figure 4E), equimolecular concentrations of enzyme and coenzyme were the most suitable choice to overcome the technical limitations for the temperature-dependent studies. Nonetheless, since K_d_^NADD^ may differ from K_d_^NADH^, the KIEs obtained in this way might be apparent. However, this does not seem to be the case, since the K_d_^NADD^ at 6 °C is quite similar to that of K_d_^NADD^ (12 ± 2 µM vs. 16 ± 3 µM, Figure 4E), indicating that, at this temperature, the deuterated substrate hardly influences this parameter. Moreover, the comparison of the limiting k_DT1_ of 122 ± 5 s ^−1^ with k_HT1_ (284 ± 17 s^−1^) resulted in a moderate value of 2.3 ± 0.5 for the KIE_A→B_ at 6 °C. On their side, ^DT^k_obsB→C_ values were considerably lower (Figure 4E, red open circles) with a limiting k_DT2_ ~ 7 s^−1^ and therefore a KIE_B→C_ may also be close to a value of 2. KIEs usually exhibit maximal values at lower temperatures, suggesting this might be a nearly limiting value. The magnitude of primary H/D KIEs theoretically can reach a maximum value of 8, although there are considerably larger values reported for some enzymes [78]. Nonetheless, primary KIEs can also decrease towards 1 when the C-H bond is either broken *less* (i.e., earlier transition state) or *more* (i.e., later transition state) in the transition state structure (asymmetrical), or if the transition state is nonlinear [79,80]. This suggests that, in the NQO1 reductive half-reaction, the transition state, at least for the first HT, is either moving away from symmetrical or it is non-linear.

**Figure 9:**
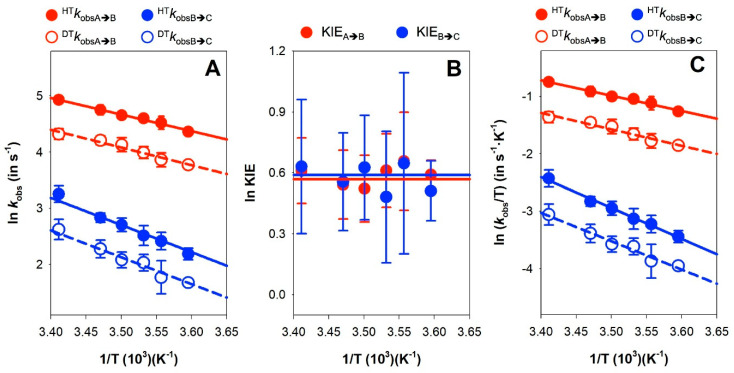
Temperature dependence of kinetic parameters for the two hydride/deuteride transfer (HT/DT) processes from NADH to NQO1. (**A**) Arrhenius plots of kinetic constants. (**B**) Temperature dependence of the kinetic isotope effects (KIEs). (**C**) Eyring plots of kinetic constants.

The magnitude of the KIE is itself informative, but the size of its temperature dependence also serves as a key descriptor of the reaction coordinate [64]. In particular, we applied the environmentally coupled tunneling model by determining HT and DT observed rate constants, namely ^HT^k_obsA→B_, ^HT^k_obsB→C_, ^DT^k_obsA→B_ and ^DT^k_obsB→C_, at different temperatures (Figure 9A). As indicated above, we used equimolecular concentrations of the enzyme and the coenzyme substrate (instead of saturating) to avoid entering the detection limit of the instrument upon increasing the temperature. The determined k_obs_ values provided apparent KIE_A→B_ and KIE_B→C_ values of ~1.8 (Table 2), which were temperature independent (Figure 9B). Despite these values being apparent, they match with the limiting one obtained under saturating conditions and low temperature. ^HT^k_obsA→B_ and ^DT^k_obsA→B_ showed a weaker temperature dependence than ^HT^k_obsB→C_ and ^DT^k_obsB→C_, and in both cases the Arrhenius plots for HT and DT hardly deviated from parallel lines, indicating very similar E_a_ values (Figure 9A). E_a_ values are moderate for the fast HT/DT event and 1.5 times larger for the second process, while ΔE_a_ (E_aDT_ − E_aHT_) is nearly 0 for both processes. In addition, the calculated isotope effect on the Arrhenius frequency factor (A_H_/A_D_) strongly differed between both HT/DT events, with a value close to the unity for the faster one and about 10 for the slower one. As a consequence of the marginal differences between E_aDT_ and E_aHT_ and the small values for the apparent KIEs, the activation enthalpies and entropies for HT and DT are also small when these results are analyzed in the context of the Eyring equation (Equation (4), see Figure 9C and Table 3).

The temperature independence of the KIE is generally interpreted in the context of full tunneling models, where the reaction barrier is attributed to the heavy atom motions that affect the probability of wave function overlap and little, or no, sampling of the distance of the reacting atoms [81]. Thus, our data show that both HT events in NQO1 are consistent with transitions under the barrier (i.e., quantum tunneling) and with asymmetrical or non-linear transition states. Moreover, the lower E_a_ and close to zero ΔE_a_ values and, particularly, the close to unity A_H_/A_D_ ratio for the fast HT process support for this event some contribution of dynamics, and/or donor–acceptor distance (DAD) fluctuations of the active site heavy atoms, to the tunneling. Thus, the fast HT process resembles the behavior most commonly found in native enzyme-mediated HT processes, in which catalytic enhancement is achieved by promoting and optimizing vibrations in active sites that minimize DAD fluctuations [81,82]. Conversely, the second HT event also shows almost temperature-independent KIEs and still similar E_aD_ and E_aH_ values, but higher E_a_ and A_H_/A_D_ considerably greater than the unity (Figure 9, Table 2). The higher E_aH_ values suggest larger reorganization energies as the main source of the E_a_, whereas the rest of the parameters, particularly the A_H_/A_D_ ratio, support a larger contribution to the active site environment to promote a close approach between the hydride donor and the acceptor atom with little DAD sampling. Altogether, these data indicate that for the slower HT process the initial pre-organization complex situates the reacting atoms, N5 of FAD and the C4-H of the nicotinamide of NADH, at optimal tunneling distance, creating a stiffer active site for the competent HT when compared to the active site of the fast HT process.

## 4. Conclusions

Our understanding of the roles of the multifunctional NQO1 protein in many physiological and pathological states, particularly in those associated with oxidative insults such as cancer and neurological disorders, is growing steadily [1,2,4,16]. In addition, NQO1 is an excellent example of an oligomeric human protein in which functional ligands exert remarkable cooperative effects with important implications for the understanding of how human genetic variability, divergent evolution and post-translational modifications shape the complex functional chemistry of multifunctional proteins [2,28,31,34,42,50,51,54,55,56,57,83]. We provide here a kinetic analysis of the oxidoreductase cycle of NQO1 in unprecedented detail. One of the main conclusions of our study is the existence of non-equivalent active sites in the protein dimer that differ in activity by about 20-fold and, thus, this could explain previous reports on the negative binding cooperativity towards inhibitors such as Dic [48,49]. At this point, we must note that in a *thermodynamic* sense, the existence of two active sites with different efficiencies (i.e., non-identical active sites) cannot be distinguished from two active sites displaying negative cooperativity (i.e., identical and non-independent active sites) [32,84]. We also report details on chemical aspects of the NQO1 catalytic cycle, such as the contributions to HT from quantum tunneling, and asymmetric transition states, as well as conformational and vibrational dynamics along the reaction coordinate. We anticipate that this detailed kinetic analysis will be valuable for understanding the effects of naturally occurring missense variants and post-translational modifications in NQO1, as well as in the rational design of novel inhibitors targeting NQO1 activity and biomacromolecular interactions [2,4].

Considering previous equilibrium binding and kinetic studies [31,48,49,54], the strong evidence provided in this work for the existence of non-equivalent active sites in the human NQO1 is not striking. Thus, our work helps to reconcile previous binding and steady-kinetic analyses focused on NAD(P)H and Dic. By looking at the NAD(P)H coenzyme, we have found strong evidence for HT occurring with a 20-fold difference in rate constants between active sites, although the fast process accounts for 75–80% of the overall HT process. This could imply that the fast pathway for HT using this coenzyme dominates the observed steady-state kinetics, thus contributing to an explanation for the lack of negative cooperativity observed in coenzyme dependence studies by steady-state kinetics [31]. However, we observed that about half of the HT process occurred through *fast* and *slow* pathways in the presence of Dic (that slowed down both pathways by three orders of magnitude). These results indicate that Dic binding causes a larger kinetic uncoupling between the two active sites, thus contributing to an explanation for the negative, but *not extreme*, cooperativity observed in inhibition studies (with Hill coefficients of about 0.5, corresponding to a cooperative binding Gibbs energy of about 1.5 kcal·mol^−1^; [48,49]). Interestingly, negative cooperativity between FAD binding sites of a similar magnitude is also observed in the apo-enzyme [54]. A detailed structural explanation for these observations, and the derived mechanistic implications, is difficult to provide for several reasons. If we consider that the two active sites *intrinsically differ* (i.e., non-identical and independent active sites), high-resolution structural information of the holo-enzyme with and without bound Dic would have revealed such structural differences between the monomers in the dimer. However, this has not been the case to the best of our knowledge (of course, we can always suggest that the reported technical aspects of the X-ray crystallography experiments, such as low temperature data acquisition, the refinement approaches used and the fact that the protein is not in solution, might have contributed to *hiding* such structural heterogeneity) [36,46]. If negative cooperativity exists (i.e., identical and dependent active sites), the clue would reside in the properties of the half-ligated species (i.e., the holo-enzyme dimer with one NAD(P)H/Dic binding site occupied) [42]. However, again, we have no structural information on these half-ligated states. However, recent molecular dynamics (MD) simulations and hydrogen-deuterium exchange (HDX) analyses have provided some clues to the structural basis of these cooperative effects [42,48]. MD simulations combined with a Gaussian network model have supported that, in the half-ligated species, the binding of Dic to one site might trigger changes in dynamics that propagate to the (empty) binding site of the adjacent monomer [48]. In addition, the holo-protein in solution shows a remarkably complex behavior in terms of local stability, supporting that, in the absence and the presence of bound Dic, different conformational substates with widely different local stability (and plausibly, intrinsic binding affinities for this inhibitor) may also be populated [42].

The kinetic heterogeneity described in this work for the two active sites in NQO1 has been further characterized by analysis of the KIEs for this enzymatic reaction. Although we must be cautious in the interpretation of these potentially apparent KIEs, according to current models of HT [64,81], these analyses supported different contributions from quantum tunneling, conformational dynamics and molecular vibrations for the fast and the slow HT processes catalyzed by NQO1. For instance, the activation enthalpies and entropies for the fast and slow pathways clearly differed, showing that the fast pathway must overcome a larger activation entropic component (Table 3). Importantly, as indicated by one of the reviewers, more robust conclusions will be drawn when several technical issues are overcome to yield the values of intrinsic KIEs in NQO1. However, at this point, it is important to keep in mind that the overall enzyme dynamics are substantially altered (not only at the active site) when Dic, the competitive inhibitor of NADH, binds to the enzyme [42] (Figure 1B). In addition, the conformational dynamics of the holo-enzyme (and to a lower extent when Dic is bound) is significantly altered by disease-associated and artificial mutants of NQO1 [30,33,50,55], as well as by phosphorylation at S82 [57], and these effects are long range (i.e., dynamic alterations are observed at regions far from the perturbed site). Therefore, the methodology presented here will pave the way to dissect at the molecular level how the dynamic alterations caused by these single-site perturbations may differently affect the fast and slow HT processes catalyzed by NQO1.

## Figures and Tables

**Table 1:** Summary of observed rate constants (*k*_obs_) for the reductive and oxidative half-reactions involving NQO1. Measurements were carried out in 20 mM HEPES-KOH, pH 7.4 at 6 °C and the ratios are indicated between brackets for each reactant. Evolution of the reaction was followed in the 400–1000 nm wavelength range using stopped-flow equipment with a photodiode array detector (n > 3, mean ± SD).

Sample in Tonometer 1	Sample in Tonometer 2	*k*_obsA→B_ (s^−1^)	*k*_obsB→C_ (s^−1^)
NQO1 (1)	NADH (1)	78 ± 1	8.9 ± 0.9
NQO1 (1)	4R-NADD (1)	44 ± 2	6.3 ± 0.2
NQO1 (1)	NADPH (1)	261 ± 13	7.8 ± 0.3
NQO1 (1) + Dic (1)	NADH (1)	0.034 ± 0.003	0.0065 ± 0.0005
NQO1 (1) + Dic (1)	NADH (6.6)	0.036 ± 0.002	0.010 ± 0.001
NQO1 (1) + Dic (4)	NADH (1)	0.018 ± 0.003	0.0015 ± 0.0001
NQO1 (1) + Dic (1)	NADPH (1)	0.036 ± 0.006	0.0070 ± 0.0008
NQO1 (1) + Dic (4)	NADPH (1)	0.017 ± 0.001	0.0020 ± 0.0001
NQO1 (1) + NADH (1)	DCPIP (1)	>500	160 ± 14
NQO1 (1) + NADH (1)	DCPIP (1) + Dic (1)	38 ± 3	6.3 ± 1.2
NQO1 (1) + NADH (1)	DCPIP (1) + Dic (4)	7.7 ± 0.2	1.3 ± 0.1
NQO1 (1) + NADH (1)	Ferricyanide	219 ± 12	29 ± 4

**Table 2:** KIEs for the HT in the reduction of NQO1 by NADH. All values correspond to data obtained with equimolecular concentrations of the reactants in the stopped-flow equipment. (n > 3, mean ± SD). Analysis was performed using Equations (2) and (3) (see Figure 9A,B).

	HT			DT			KIE	ΔE_a_ E_aDT_ − E_aHT_ (kcal·mol^−^^1)^	A_H_/A_D_
	^HT^*k*_obs_^a^ (s^−1^)	E_aHT_ (kcal·mol^−^^1^)	A_H_ (s^−1^)	^DT^*k*_obs_^a^ (s^−1^)	E_aDT_ (kcal·mol^−^^1^)	A_D_ (s^−1^)			
A→B	78 ± 1	6.1 ± 0.2	(5.3 ± 1.2)·10^6^	44 ± 2	6.3 ± 0.4	(4.1 ± 1.1)·10^6^	1.8 ± 0.1	0.2 ± 0.4	1.3 ± 0.6
B→C	8.9 ± 0.9	10.9 ± 0.5	(3.4 ± 0.9)·10^9^	5.3 ± 0.2	9.8 ± 0.5	(2.6 ± 0.6)·10^8^	1.8 ± 0.3	−1.1 ± 0.7	13 ± 6

^a^ Values at 6 °C.

**Table 3:** Activation enthalpies (ΔH^‡^) and entropies (ΔS^‡^) obtained using the Eyring equation (Equation (4)) and temperature-dependent *k*_obs_ values (see Figure 9C).

Activation Parameters	HT		DT	
	A→B	B→C	A→B	B→C
ΔH^‡^ (kcal·mol^−1^)	5.3 ± 0.3	11 ± 1	5.7 ± 0.6	9.8 ± 0.8
ΔS^‡^ (cal·mol^−1^·K^−1^)	−31 ± 1	−16 ± 2	−30 ± 2	−20 ± 3

**Figure A1:**
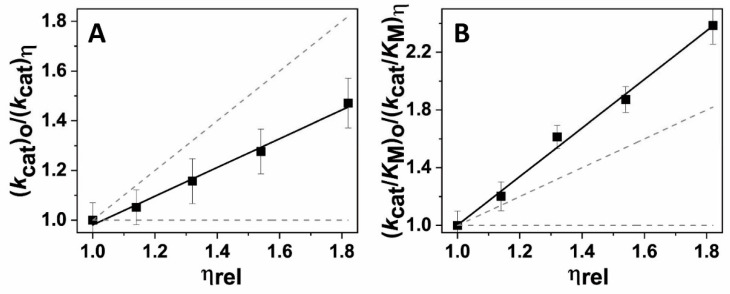
Viscosity dependence of NQO1 steady-state parameters. (**A**) Plot of (*k*_cat_/*K*_m_)_o_/(*k*_cat_/*K*_m_)_η_ vs. η_rel_. (**B**) (*k*_cat_)_o_/(*k*_cat_)_η_ vs. η_rel_. Dashed lines guide the eye along slopes with values of 1 and 0, respectively.

## Appendix A. Kinetic Solvent Viscosity Effects (KSVEs)

As pointed out by one of the reviewers, the role of additional steps, such as substrate binding/product release as a rate-limiting step, should be considered. To this end, we analyzed the so-called *kinetic solvent viscosity effects* (KSVEs) on the steady-state parameters of NQO1. [85]. The diaphorase activity of NQO1 was evaluated using DCPIP as the electron acceptor at different concentrations of glycerol as a viscogen. Reaction mixtures contained 20 μM DCPIP, 1 nM recombinant NQO1 and NADH at varying concentrations at 25 °C. The reaction was triggered by the addition of the enzyme and followed by absorption changes at 600 nm for 1 min in 1-cm path length quartz cuvettes in a thermostatized Cary 100 spectrophotometer (Agilent). The specific activity was calculated using ε_620nm_ = 21,000 M^−1^·cm^−1^ for DCPIP. Kinetic parameters were determined by varying the NADH concentration [34]. The *k*_cat_ and *K*_M_ values were determined using the Michaelis–Menten equation.

A plot of (*k*_cat_)_o_/(*k*_cat_)_η_ vs. relative viscosity (η_rel_) yields a slope below unity (0.55 ± 0.05) (Figure A1), indicating that product(s) release could be partially limiting the enzyme turnover rate. A plot of (*k*_cat_/*K*_M_)_o_/(*k*_cat_/*K*_M_)_η_ vs. η_rel_ yields a slope over 1 (1.6 ± 0.1) (Figure A1), thus supporting that the diffusion of substrates is not relevant and that the viscogen could manifest inhibitory behavior due to effects on structural dynamics (e.g., in protein loops).
